# Supplementary material for: scGEM: Unveiling the Nested Tree-Structured Gene Co-Expressing Modules in Single Cell Transcriptome Data
Source: Cancers (Basel). 2023 Aug 26;15(17):4277. doi: 10.3390/cancers15174277 (PMC10486867; doi:10.3390/cancers15174277)
Supplement: Supplementary file 1 [file cancers-15-04277-s001.zip › cancers-2543401-supplementary.pdf]

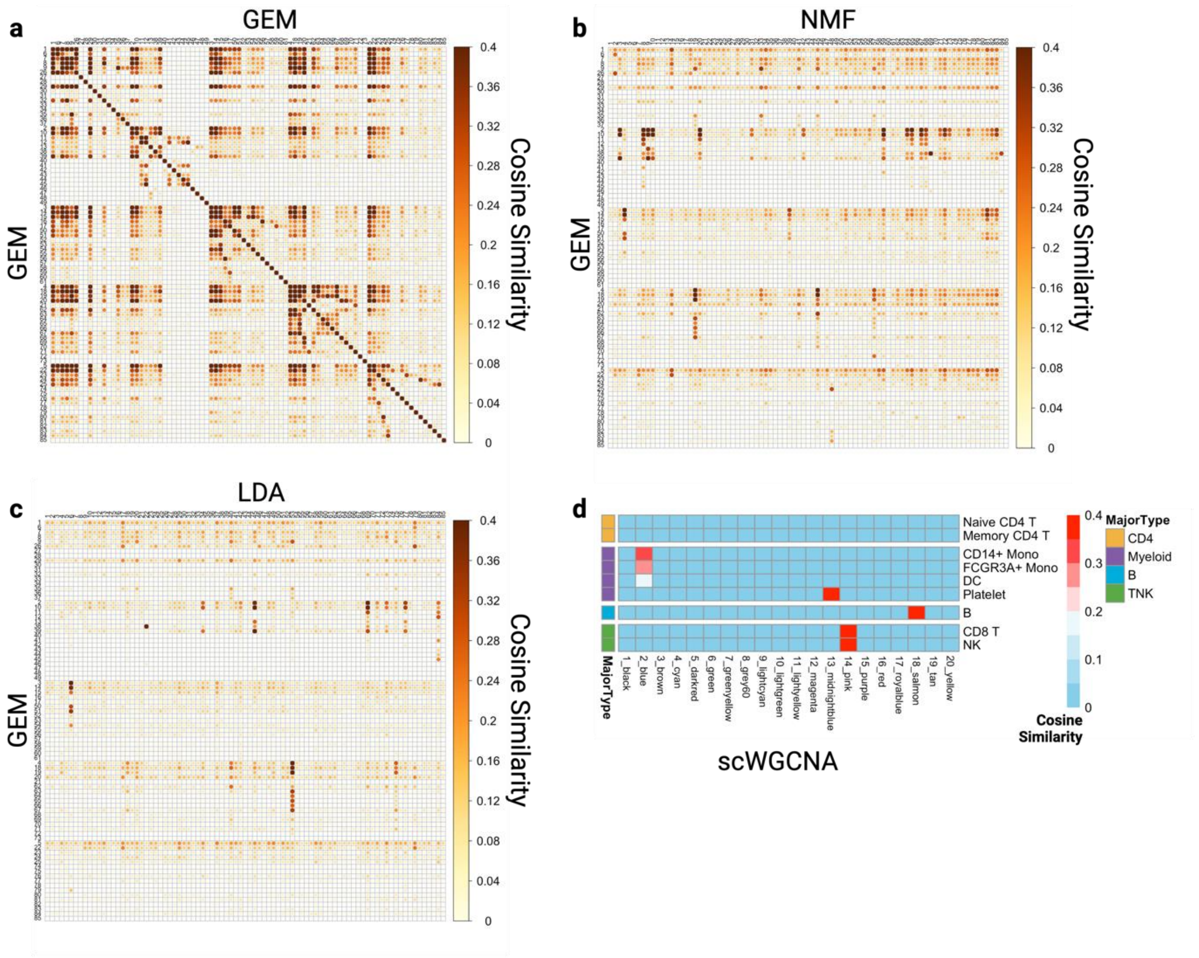

Figure S1. Module comparisons in PBMC 3K dataset. (**a**) Pairwise correlation among GEMs, measured in cosine similarity of the top 150 weighted genes in each GEM. (**b**) Pairwise correlation between scGEM and modules factorized in NMF method, measured as the same in (a). (**c**) Pairwise correlation between scGEM and modules learning in LDA method using cosine similarity. (**d**) Cosine simlarity between the module genes in scWGCNA and highly variable genes in each cell subtype. In total, 20 modules were found.

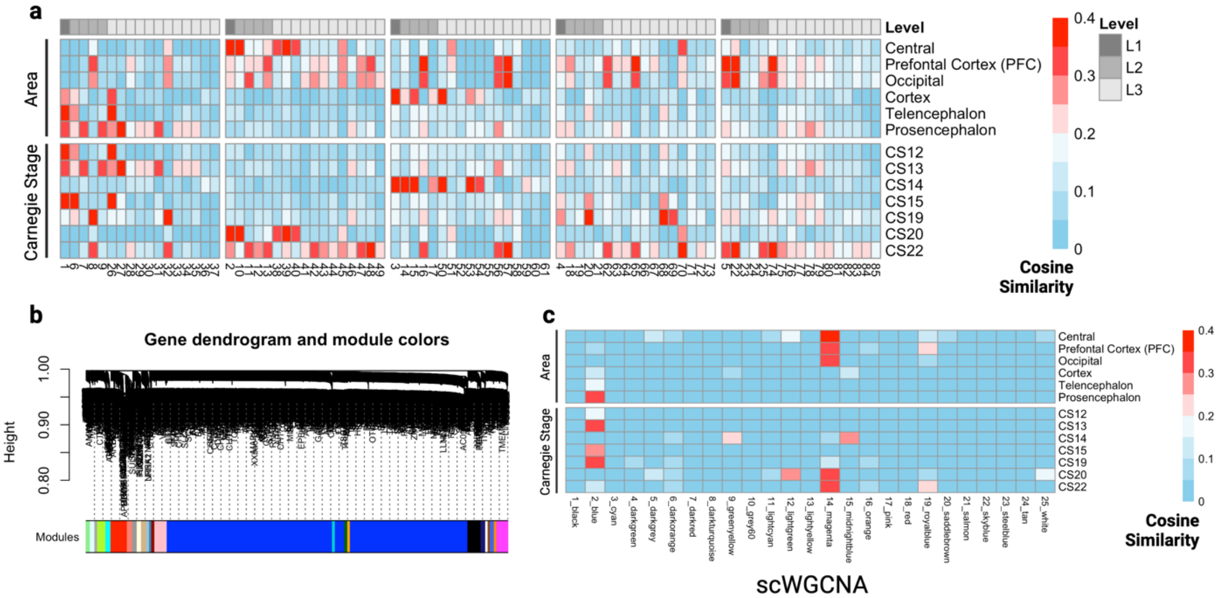

Figure S2. scGEM and scWGCNA results in early human brain development single cells. (**a**) GEM distribution in each annotated area and Carnegie stage (CS), measured by cosine similarity. GEM is noted with respect to associated node level. (**b**) scWGCNA output, there are 25 modules that were detected. (**c**) Relationship between modules in scWGCNA and annotated area and Carnegie stage. Relationship was measured based on the module membership genes and differentially expressed genes in each cell cluster.
